# Search for biomarkers of asbestos exposure and asbestos-induced cancers in investigations of the immunological effects of asbestos

**DOI:** 10.1186/s12199-017-0661-4

**Published:** 2017-06-09

**Authors:** Hidenori Matsuzaki, Naoko Kumagai-Takei, Suni Lee, Megumi Maeda, Nagisa Sada, Tamayo Hatayama, Shoko Yamamoto, Miho Ikeda, Kei Yoshitome, Yu Min, Yasumitsu Nishimura, Takemi Otsuki

**Affiliations:** 1 Department of Hygiene, Kawasaki Medical School, 577 Matsushima, Kurashiki, 701-0192 Japan; 2 Department of Biofunctional Chemistry, Division of Bioscience, Okayama University Graduate School of Natural Science and Technology, 1-1-1 Tsushimanaka, Kita-Ku, Okayama 700-8530 Japan; 3 Department of Occupational Diseases, Zhejiang Academy of Medical Sciences, 182 Tian Mu Shan Road, Zhejiang, 310013 People’s Republic of China

**Keywords:** Asbestos, Immune cells, Biomarker, Cytokine, Cell surface marker, Tumor immunity

## Abstract

The immunological effects of asbestos exposure on various lymphocytes such as the regulatory T cell (Treg), responder CD4+ T helper cell (Tresp), CD8+ cytotoxic T lymphocytes (CTL), and natural killer (NK) cells were investigated. Results show that asbestos exposure impairs antitumor immunity through enhancement of regulatory T cell function and volume, reduction of CXCR3 chemokine receptor in responder CD4+ T helper cells, and impairment of the killing activities of CD8+ cytotoxic T lymphocytes (CTL) and NK cells. These findings were used to explore biological markers associated with asbestos exposure and asbestos-induced cancers and suggested the usefulness of serum/plasma IL-10 and TGF-β, surface CXCR3 expression in Tresp, the secreting potential of IFN-γ in Tresp, intracellular perforin level in CTL, and surface expression NKp46 in NK cells. Although other unexplored cytokines in serum/plasma and molecules in these immunological cells, including Th17, should be investigated by experimental procedures in addition to a comprehensive analysis of screening methods, biomarkers based on immunological alterations may be helpful in clinical situations to screen the high-risk population exposed to asbestos and susceptible to asbestos-related cancers such as mesothelioma.

## Background

Asbestos exposure causes various benign and malignant diseases [1–5]. It induces one of the most typical forms of pneumoconiosis, known as asbestosis, as a consequence of relatively high doses of exposure. This condition is basically lung fibrosis [6, 7]. Immune competent cells play an important role when asbestos fibers first enter the human body, such as alveolar macrophages that act against these foreign materials by activating the NOD-like receptor family pyrin domain containing 3 (NLRP3: NALP3) inflammasome to produce interleukin (IL)-1β and attract fibroblasts [8, 9]. Patients with asbestosis suffer from various respiratory symptoms caused by lung fibrosis, including shortness of breath, cough, sputum, and symptoms of right-sided heart failure at the advanced stage. In addition to asbestosis, asbestos exposure causes benign pleural diseases such as pleural plaque (PP) and diffuse pleural thickening (DPT). Although patients with PP do not show any progression of respiratory symptoms, those with DPT at the advanced stage suffer from severe respiratory failures [1–5].

Moreover, asbestos exposure is known to cause malignant diseases such as lung cancer and malignant mesothelioma (MM), as well as other cancers such as those of the ovary, pharynx, and larynx [1–5]. MM remains an incurable cancer, and therefore, the ability to detect the very early stage of MM would be very important for its treatment and possible cure [10–12].

There are various candidates for biomarkers of MM as tumor markers. However, most of the tumor markers are derived from molecules produced and secreted from mesothelioma cells. This means that when the tumor mass is relatively small, the levels of some tumor markers in serum might be low and then gradually increase as the tumor develops [13–18].

The importance of these disease/tumor markers is their potential use to screen the high-risk population exposed to asbestos. This population includes the recent and past workers in asbestos-handling factories, family members of these workers, people who have a history of residing near these factories, and workers employed in building demolition, as well as rubble processing workers utilized after various natural and man-made disasters such as an earthquake.

A single biomarker is not suitable to detect the occurrence of MM in these populations. Therefore, radiological screening is used to detect PP or other findings in chest X-rays or computed tomography of the chest. However, the use of radiological methods for screening involves important problems such as high cost and further exposure to radiation. Moreover, a screening frequency limited to once every 6 or 12 months is not sufficient to detect the early stage of MM [19–21].

The immunological effects of asbestos on the various circulating immune cells have not been investigated thoroughly because the main target of asbestos fibers are thought to be lung epithelial cells and pleural mesothelial cells in regard to carcinogenesis, as well as immune cells such as alveolar macrophage located at lesions of asbestos-entering sites. However, silica particles, comprising Si and O_2_ and which represent the core components of asbestos fibers chemically, cause alteration of immune cells in various autoimmune diseases [22–24]. Although the physiological effects caused by silica and asbestos differ, including those produced by fibers and particles, asbestos fibers may influence the various circulating immune cells in a manner similar to silica particles. In particular, the immunological effects of asbestos may be the reduction of antitumor immunity as evidenced by malignant complications of asbestos exposure detected after a long latent period such as 30 to 40 years for MM [25–27].

Thus, we have been investigating the immunological effects of asbestos on various human immune cells such as responder T cells (Tresp), regulatory T cells (Treg), CD8+ cytotoxic T lymphocytes (CTL), and natural killer (NK) cells. Tresp cells are defined as CD4+CD25− T helper cells which are able to proliferate by antigen stimulation. It is commonly considered as memory T helper cells. Although these investigations aimed to explore the immunological effects of asbestos, we found that some results could be utilized to develop immunological screening methods for asbestos exposure and the occurrence of mesothelioma. In this review, findings regarding the immunological effects of asbestos are introduced, and a method for constructing immunological biomarkers for asbestos exposure and detection of mesothelioma is discussed.

### T helper cells

To investigate the effects of asbestos exposure on human T cells, a cell culture model for continuous low-dose exposure that simulated human exposure was established using a human polyclonal T cell line immortalized by human T cell leukemia/lymphoma virus 1 (HTLV-1), designated as MT-2 [28, 29]. In the initial screening to select the cell line, various virus immortalized and tumor cell lines derived from human T and B cells were exposed to chrysotile asbestos fibers. Among the cell lines including MT-2, T cell tumors [MT-1; adult T cell leukemia (ATL), Molt-4, CEM, and Jurkat; T cell acute lymphoblastic leukemia (T-ALL)], and B cell lines [Epstein-Barr virus immortalized B cell lines (KMS-9 and KMS-15), Raji; Burkitt’s lymphoma, SUDHL-4; B cell lymphoma, KMM-1; myeloma], the MT-2 cell line was most sensitive for growth inhibition [30]. MT-2 was selected because it was better to use an immortalized cell line not derived from tumor cells and a T cell line rather than B cells to explore the immune effects. Since the MT-2 cell line included CD4+ cells and transient exposure to chrysotile resulted in production of reactive oxygen species (ROS) and activation of the mitochondrial apoptotic pathway [28], cells were initially exposed continuously to chrysotile at a concentration that induces apoptosis in less than half of the cells. These cells were then monitored monthly for the appearance of apoptosis following the transient and high-dose exposure to chrysotile. After approximately 1 year of continuous exposure, an MT-2 subline showed resistance to chrysotile-induced apoptosis [29]. This indicated that cell features were changed by continuous exposure to asbestos. Independent sublines exposed to chrysotile or crocidolite were established. cDNA microarray analysis of these sublines showed similar patterns, which differed from those of the MT-2 original cells [31].

The typical features of these sublines that differed from the original cells included (1) increased production of IL-10 and transforming growth factor (TGF)-β [29, 32], (2) reduced expression of cell surface C-X-C chemokine receptor type 3 (CXCR3) chemokine receptor [31, 33], (3) reduced expression of intracellular interferon (IFN)-γ [31, 33], and (4) increased expression of Bcl-2, anti-apoptotic molecule [29]. The decreased expression of CXCR3 and higher expression of Bcl-2 were confirmed in peripheral blood CD4+ (mainly Tresp population) cells derived from PP and MM [29]. These changes were greater in cases involving MM compared to PP. The findings indicated that these cellular and molecular changes were caused by asbestos exposure and advanced when mesothelioma occurred.

The MT-2 cell line was found to possess Treg function. Thus, Treg function in MT-2 original cells and sublines exposed continuously to asbestos was useful because of the excess production of IL-10 and TGF-β [29, 32], which are known to be typical soluble factors secreted from Treg cells to inhibit the proliferative reaction of Tresp against foreign antigens [34–36]. The Treg inhibitory function was enhanced in continuously exposed sublines when compared with that of the MT-2 original cell line by cell-cell contact. In addition, knockdown of IL-10 or TGF-β using the siRNA method resulted in reduction of the inhibitory function of the sublines [37]. Moreover, continuously exposed sublines showed reduced expression of Forkhead box protein O1 (FoxO1) transcription factor [38]. FoxO1 is known to positively regulate breaking cell cycle regulators such as cyclin-dependent kinase inhibitors (CDK-I; ink4 families and Cip/Kip families) and negatively regulate accelerating factors such as cyclins. Reduced expression of FoxO1 in continuously exposed sublines was associated with markedly enhanced cyclin D1 expression compared with that in MT-2 original cells, and various CDK-Is exhibited reduced expression [39]. In addition, knockdown of FoxO1 in MT-2 original cells resulted in enhancement of cyclin D1 expression. Moreover, the cell cycle progressing index represented by [S phase cell number divided by G1 phase cell number] increased in continuously exposed sublines relative to that of MT-2 original cells [39]. All of these findings indicated the asbestos-exposed Treg exhibited enhanced function and increased numbers.

### NK cells

Similar to the aforementioned investigation of T helper cells, the effects of asbestos fibers on NK cells were studied using the NK cell line YT-A1 [40]. After more than 5 months of culture with chrysotile asbestos, a continuously exposed subline showed reduced killing activity against K562 target cells, derived from a human erythromyeloblastoid leukemia cell line commonly used to examine the killing activity of human NK cells. A YT-A1 subline exposed continuously to chrysotile showed decreased cell surface expression of NKG2d and 2B4 activation receptors, as well as a reduced intracellular perforin level compared with original YT-A1 cells which were never exposed to asbestos. Additionally, signal transduction leading to the phosphorylation of extracellular signal-regulated kinases (ERKs) 1 and 2 in the mitogen-activated protein kinase (MAPK) pathway was impaired in the YT-A1 subline due to decreased expression of NKG2D [41].

We also examined the NK cell killing activity from freshly isolated peripheral blood mononuclear cells (PBMC) derived from healthy volunteers (HV), patients with PP, and those with MM [40–43]. Results revealed reduced killing activity in asbestos-exposed patients with PP and MM. The surface expression levels of another NK cell activating receptor, NKp46, were significantly correlated with killing activities [40–43]. These results indicated that asbestos exposure reduces the expression of NK cell activating receptor and thereby causes impairment of killing activity. These findings emphasized the importance of the NKp46 expression level in NK cells of human peripheral blood.

### CTL

The effects of exposure to asbestos fibers on another important lymphocyte, CTL, were also examined in regard to antitumor immunity. Initially, the effects of chrysotile following in vitro induction of CTL by the mixed lymphocyte reaction (MLR) using PBMCs from HV with irradiated allo-PBMCs were estimated in relation to CD8+ T cells [44]. Clonal expansion represented by proliferation of CD8+ cells and differentiation to CTL from CD8+ cells defined by killing activity against allo-PBMCs, increase of CD45RO and decrease of CD45RA surface markers, increase of CD25 as an activation marker, and production of IFN-γ were all impaired when MLR was performed with addition of chrysotile asbestos. Furthermore, IL-2 in the supernatants did not change by much at day 4 and day 7 during MLR with asbestos [44]. Since IL-2 possesses an important role for clonal expansion of CTL, we examined whether supplementation with IL-2 rescued the impairment of clonal expansion of CTL. Results showed that although supplemented IL-2 did not increase the number of CD8+ cells or rescue changes in CD45RA, RO, and CD25 expression, the killing activity was recovered. Part of this recovery is explained by the increase of intracellular granzyme B in CD8+ cells, particularly in the proliferating cells [45]. These results indicated that asbestos disturbs the clonal expansion of CTL. The supplemented IL-2 was not sufficient to recover the impaired clonal expansion. Thus, other cytokines such as IL-15 or stimulatory molecules bound on the CTL cell surface may be important.

We investigated the functional properties of CD8+ lymphocytes in patients with PP and MM. CD8+ cell status was examined using peripheral blood derived from these patients. Although the total numbers of PBMCs from PP and MM were lower than those from HV, the percentage of CD8+ cells did not differ between HV, PP, and MM. In addition, IFN-γ+ and CD107a+ (as the marker of degranulation and the secretion of cell attacking molecules such as granzymes and perforin) cells in phorbol 12-myristate 13-acetate (PMA) and ionomycin-stimulated CD8+ cells did not differ between these three groups [45]. However, the percentage of granzyme B+ and perforin+ cells in PMA/ionomycin stimulated CD8+ cells was higher in the PP group compared with HV. The MM group showed a decrease of the perforin level in CD8+ cells after stimulation compared with that of the PP group. These results indicate that although asbestos-exposed patients such as those with PP and MM possessed the common character of functional alteration in CTL defined as an increase of memory cells, CTL in MM exhibited impaired stimulation-induced cytotoxicity [46–48]. This was not observed in CTL from PP.

### Explore biomarkers of asbestos exposure and occurrence of MM

**Fig. 1:**
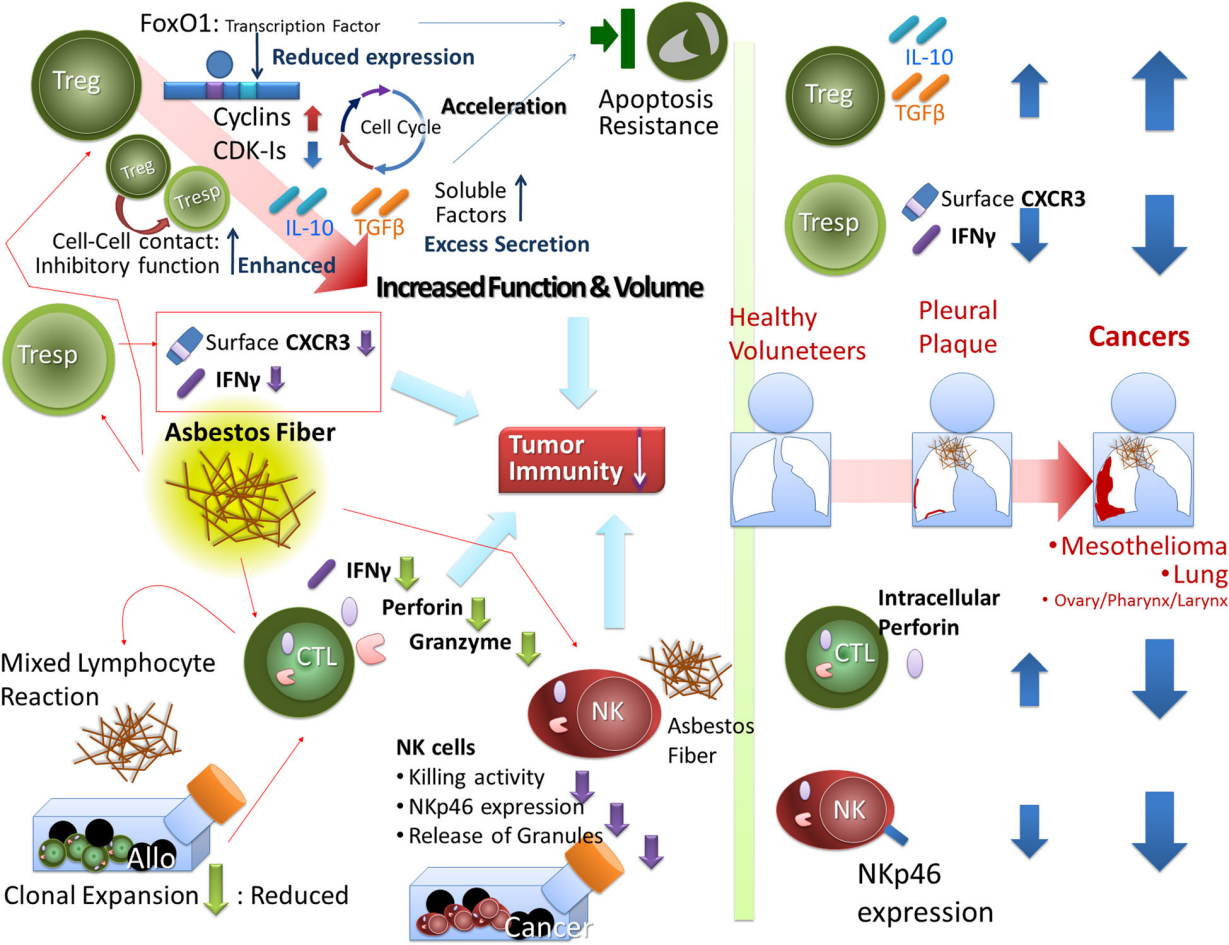
Schematic presentation of the immunological effects of asbestos exposure on various lymphocytes such as regulatory T cells (*Treg*), responder CD4+ T helper cells (*Tresp*), CD8+ cytotoxic T lymphocytes (*CTL*), and natural killer (*NK*) cells. All of the findings described in this review indicate that asbestos exposure impairs antitumor immunity, as shown in the *left panel* of the figure. These findings can be used to explore biological marker candidates, as shown in the *right panel* of the figure, and suggest the usefulness of serum/plasma IL-10 and TGF-β, surface CXCR3 expression in Tresp, secreting potential of IFN-γ in Tresp, intracellular perforin level in CTL, and surface expression of NKp46 in NK cells. Although other unexplored cytokines in serum/plasma and molecules in these immunological cells and Th17 should be investigated, including a comprehensive analysis of screening methods, biomarkers based on immunological alterations may helpful in the clinical situation to screen the high-risk population exposed to asbestos and to detect and treat asbestos-related cancers such as mesothelioma

This review has outlined the many changes regarding immunological status in asbestos-exposed patients such as those with PP and MM. As shown in the left part of Fig. 1, all of these changes indicated that asbestos exposure causes impairment of antitumor immunity, such as increased function and volume of Treg, decreased CXCR3 and IFN-γ in Tresp, impairment of NK cell killing activity with reduction of surface NKp46 expression, and disturbance of CTL function and numbers with perturbation of killing molecules such as perforin and granzymes.

Although a single marker may not be able to detect previous or present asbestos exposure, or the occurrence of MM, the studies detailed in this review indicate it would be possible to combine several markers, such as serum/plasma IL-10 and TGF-β concentrations, cell surface expression level of CXCR3 in CD4+ cells, secreting potential of IFN-γ in CD4- or CD8-positive cells, intracellular expression of perforin in CD8+ cells, and the surface expression of NKp46 in NK cells, as shown in the right panel of Fig. 1. Moreover, it may be possible to examine the mRNA expression levels of these molecules, particularly the lymphoid cell type. In order to use immunological biomarkers or an immunological formula to detect asbestos exposure and/or the occurrence of MM, a standardized method must be employed regarding how venous-drawn peripheral blood is divided, for example, into plasma and lymphocytes (or into CD4+, CD8+ cells, and NK cells). Additionally, it is necessary to examine mRNA expression and molecules expressed intracellularly in various lymphocyte subgroups. In addition, comprehensive analyses of various cytokines in plasma/serum from asbestos-exposed patients such as those with PP and MM in comparison to HV should be performed to detect other cytokines as biomarker candidates, as reported previously. Moreover, the status of function and volumes of the Th17 subtype of helper T cells should be investigated since the conversion and polarization of Treg and Th17 depends on the cytokine status surrounding these cells such as IIL-6 and TGF-β [49–52].

If a formula or combined biomarkers based on immunological alteration caused by asbestos exposure are obtained, the clinical advantages would include their ease of use compared to current screening for asbestos exposure using radiological methods with hazardous radiation exposure, lower costs for screening, and an increased frequency of examinations among the high-risk population exposed to asbestos at present, recently, or in the past.

## Conclusion

Investigations of the immunological effects of asbestos fibers in various human immune cells such as Treg, Tresp, NK cells, and CTL suggest biomarker candidates for the biological detection of asbestos exposure and the occurrence of MM. It may be possible to use a combination of markers or a formula representing the various changes in immune cells, including cytokines produced from these cells. Although additional investigations are necessary to detect other altered molecules in various immune cells following asbestos exposure, immunological markers are better than radiological screening in regard to costs, procedure (only require drawing peripheral blood), and possibly accuracy. Further studies of the immunological effects of asbestos exposure are required to fully explore the biological alteration induced by asbestos exposure and to develop clinical or preventive methods based on extracted markers that will reduce the suffering of asbestos-exposed patients.

## Abbreviations

ATL: Adult T cell leukemia
CDK-I: Cyclin-dependent kinase inhibitor
CTL: Cytotoxic T lymphocytes
CXCR3: C-X-C chemokine receptor type 3
DPT: Diffuse pleural thickening
ERK: Extracellular signal-regulated kinase
FoxO1: Forkhead box protein O1
HTLV-1: Human T cell leukemia/lymphoma virus 1
HV: Healthy volunteers
IFN: Interferon
IL: Interleukin
MLR: Mixed lymphocyte reaction
MM: Malignant mesothelioma
NK: Natural killer
NLRP3: NALP3NOD-like receptor family pyrin domain containing 3
PBMC: Peripheral blood mononuclear cells
PMA: Phorbol 12-myristate 13-acetate
PP: Pleural plaque
ROS: Reactive oxygen species
T-ALL: T cell acute lymphoblastic leukemia
TGF: Transforming growth factor
Treg: Regulatory T cells
Tresp: Responder T cells
